# Phosphodiesterase 5a Inhibition with Adenoviral Short Hairpin RNA Benefits Infarcted Heart Partially through Activation of Akt Signaling Pathway and Reduction of Inflammatory Cytokines

**DOI:** 10.1371/journal.pone.0145766

**Published:** 2015-12-28

**Authors:** Longhu Li, Dong Zhao, Zhe Jin, Jian Zhang, Christian Paul, Yigang Wang

**Affiliations:** 1 Department of Cardiology, the First Hospital of Qiqihaer City, Qiqihaer, China; 2 Department of Cardiology, the Affiliated Zhongshan Hospital of Dalian University, Dalian, China; 3 Collaborative Innovation Center of Judicial Civilization, China, Key Laboratory of Evidence Science (China University of Political Science and Law), Ministry of Education, Beijing, China; 4 Department of EICU, the Affiliated Zhongshan Hospital of Dalian University, Dalian, China; 5 Department of Pathology and Lab Medicine, University of Cincinnati Medical Center, 231 Albert Sabin Way, Cincinnati, Ohio, 45267, United States of America; Georgia Regents University, UNITED STATES

## Abstract

**Introduction:**

Treatment with short hairpin RNA (shRNA) interference therapy targeting phosphodiesterase 5a after myocardial infarction (MI) has been shown to mitigate post-MI heart failure. We investigated the mechanisms that underpin the beneficial effects of PDE5a inhibition through shRNA on post-MI heart failure.

**Methods:**

An adenoviral vector with an shRNA sequence inserted was adopted for the inhibition of phosphodiesterase 5a (Ad-shPDE5a) *in vivo* and *in vitro*. Myocardial infarction (MI) was induced in male C57BL/6J mice by left coronary artery ligation, and immediately after that, the Ad-shPDE5a was injected intramyocardially around the MI region and border areas.

**Results:**

Four weeks post-MI, the Ad-shPDE5a-treated mice showed significant mitigation of the left ventricular (LV) dilatation and dysfunction compared to control mice. Infarction size and fibrosis were also significantly reduced in Ad-shPDE5a-treated mice. Additionally, Ad-shPDE5a treatment decreased the MI-induced inflammatory cytokines interleukin (IL)-1β, IL-6, tumor necrosis factor-α, and transforming growth factor-β1, which was confirmed *in vitro* in Ad-shPDE5a transfected myofibroblasts cultured under oxygen glucose deprivation. Finally, Ad-shPDE5a treatment was found to activate the myocardial Akt signaling pathway in both *in vivo* and *in vitro* experiments.

**Conclusion:**

These findings indicate that PDE5a inhibition by Ad-shPDE5a via the Akt signal pathway could be of significant value in the design of future therapeutics for post-MI heart failure.

## Introduction

Chronic heart failure is the leading cause of mortality and morbidity worldwide, and is commonly a result of myocardial infarction (MI)-induced remodeling of the left ventricle (LV), which is characterized by LV dilatation and cardiac dysfunction[1, 2]. There is therefore a critical need for therapies that effectively inhibit LV remodeling and preserve cardiac function in order to improve the clinical outcome of patients after MI.

The cyclic nucleotide cGMP plays a central role in cardiovascular regulation, influencing function, gene expression, and morphology[3]. Cardiac cGMP is hydrolyzed by members of phosphodiesterase (PDE) family[4] of which PDE5a acts more specifically[5]. PDE5a is a cytosolic protein and its inhibition was reported to benefit patients with pulmonary hypertension[6]. Myocardial PDE5a expression has been shown to increase in patients with advanced heart failure and contribute to LV remodeling subsequent to myocardial infarction[7]. Several previous studies have demonstrated the beneficial effects of PDE5a chemical inhibitors on heart disease, including the commercially offered drugs sildenafil, tadalafil and vardenafil[8–10]. Our recent study focused on prolonging the effective inhibition of PDE5a, and results demonstrated that PDE5a inhibition using an adenoviral vector inserted into the shRNA sequence could improve cardiac function and remodeling[11], as well as increase capillary density and capillary/myocyte ratio in our MI mouse model.

In addition, evidence from both animal and human studies suggests that increased inflammatory cytokines are associated with a poor prognosis following MI and may play an important role in the pathogenesis and progression of heart failure. Cytokines can influence heart contractility by inducing hypertrophy and promoting apoptosis or fibrosis, thereby contributing to the continuous myocardial remodeling process [12–14]. In pelvic ganglia neurons, for instance, a recent study has shown that PDE5 inhibition attenuates inflammation and oxidative stress after bilateral cavernosal nerve damage [15].

Our aims in the present study were to confirm the beneficial effects of PDE5a inhibition with Ad-shPDE5a on chronic post-MI heart failure, investigate the role of inflammatory cytokines involved in those effects, and characterize the molecular signaling upstream of the outcomes.

## Materials and Methods

### Animal Experimental Protocols

All animal work was conducted in accordance with the Guide for the Care and Use of Laboratory Animals published by the US National Institutes of Health (NIH Publication No. 85–23, revised 1996). This study was approved by the IACUC at University of Cincinnati (Protocol Number: 06-03-03-01).

Humane endpoints during the animal survival study were identified as animals with weight loss of 20% or greater and/or animals with respiratory distress after opening of thoracic cavity surgery. If symptoms were to occur, veterinary staff was consulted and appropriate medical treatment was provided. If animals did not respond to appropriate treatment, animals were euthanized.

The animals were euthanized under anesthesia by cardioplegic solution injected into the heart to arrest the heart at diastolic phase followed by removal of vital organ.

Animals were monitored on a daily basis with assistance of the LAMS Veterinary Staff in evaluation the health of the animals. There were no unexpected deaths for the duration of the study.

Animal’s pain/distress was monitored to ensure that the analgesic is effective by watching for guarding (protecting painful area), licking, biting, scratching, or shaking the painful area, restlessness, lack of normal interest in surroundings, failure to groom, abnormal postures, and lack of mobility.

MI was induced in 10-week-old male C57BL/6J mice by ligation of the left coronary artery as previously described[16]. Immediately after that, adenoviral Ad-shPDE5a (1x10^10^ particles) was injected into multiple sites per mouse heart along the anterior and posterior left ventricular wall. As a control, adenoviral Null (Ad-Null) or DMEM culture medium (DMEM) was injected in the same manner. In sham-operated mice (Sham), the suture was passed but not tied.

PDE5a-specific shRNA was designed based on rat PDE5a sequence; two complementary oligonucleotides of PDE5a were: forward 5′-gatccggagcagcagtcattggaagtcgaaacttccaatgactgctgctccttttttg-3′ and reverse 5′-aattcaaaaaaggag cagcagtcattggaagtttcgacttccaatgactgctgctccg-3′. The double-strand oligonucleotides were ligated into RNAi-Ready pSIREN-DNR-DsRed-Express Vector (Clontech, Mountain View, CA). Then the Donor Vector was inserted into pLP-Adeno-X LP CMV Vector (Clontech, Mountain View, CA) to be constructed as the recombinant pLP-Adeno-X PDE5a shRNA (Ad-shPDE5a).The Ad vector without therapeutic shRNA was prepared as control adenovirus (Ad-Null). These replication-deficient Ad-shPDE5a and Ad-Null vectors were propagated in HEK-293 cells using Dulbecco's modified Eagle's medium (DMEM; Sigma, St. Louis, MO) supplemented with 10% fetal bovine serum (FBS, Sigma). At the stipulated time, the supernatant from HEK-293 were collected for adenovirus purification with Adeno-X Maxi Purification Kit (Clontech).

MI mice were randomly assigned to the Ad-shPDE5 (n = 12), Ad-Null (n = 15) or DMEM (n = 12) treatment group, and followed up for 4 weeks. Sham-operated mice (n = 6) were injected with the same volume of DMEM in a similar manner and examined 4 weeks later.

### Preparation and Transduction of Cultured Myofibroblasts

Myofibroblasts were isolated from 1-day-old neonatal C57BL/6J mice as reported previously[17]. Briefly, left ventricle tissue was excised, washed with HBSS and cut into pieces of 1~2 mm. Enzymatic digestion using Collagenase Type II (100 U) and 0.125% trypsin-EDTA was employed to dissociate the tissue pieces, which were agitated at 37°C for 40~60 min. Supernatant was collected every 10 min into a conical tube and digestion was neutralized with 1/10 volume of fetal bovine serum. Finally, the cells were centrifuged at 1,200 rpm for 5 min, resuspended in fresh medium (DMEM containing 10% FBS) and plated into 25 cm2 culture flasks. Non-adherent cells were discarded after 60 min. The remaining cells were propagated 4~5 passages, which then spontaneously induced into myofibroblasts. The myofibroblasts were plated on dishes and incubated in DMEM containing 10% FBS at 37°C for 24 h. The cells were exposed to infection medium containing 1x10^8^ Ad-vector particles/ml for 16 h, followed by maintenance in normal medium for 56 h. The cells were used for biochemical analyses. Proteins extracted from myofibroblasts were used for Western blotting.

Myofibroblast culture medium was replaced with glucose- and serum-free DMEM for oxygen and glucose deprivation (OGD), and the cells were kept in an airtight anoxia chamber saturated with 95% N2/5% CO2. After 4 h or 8 h of incubation under oxygen and glucose deprivation, proteins were extracted from myofibroblasts and used for Western blot and ELISA.

### Cardiac Function Studies

Animals were anesthetized via intraperitoneal injection with pentobarbital. Echocardiograms were then recorded 4 weeks post-MI, using an echocardiographic system (HDI-5000 SONOS CT) equipped with Compact Linear Array probe CL10-5. The heart was imaged in M-Mode, and recordings were obtained from parasternal long-axis view at the papillary muscle level.

### Histological Analysis

After the physiological studies were completed, all surviving mice were euthanized and their hearts were removed. For measurement of infarction size and area of fibrosis, the heart was arrested in diastole by intravenous injection of cadmium chloride and fixed using formalin. The heart was then excised, cut transversely, and embedded in paraffin. Histological sections of 4-μm thickness were cut and used for hematoxylin-eosin and Masson’s trichome staining for visualization of muscle architecture and thickness of the LV wall as described in the previous study[18]. Fibrosis and total LV area of each image were measured using the Image-Pro Plus (Media Cybernetics).

### Western blot and ELISA

Proteins from heart tissues and cultured myofibroblasts were used for Western blot analysis. Proteins were separated and transferred to membranes by standard protocols, after which they were probed with antibodies against p-Stat3, p-Stat5, p-Akt, and p-Erk (all from Cell Signaling) and transforming growth factor (TGF-β1) (Promega). Three to five hearts from each group or cultured myofibroblasts were subjected to the Western blot. The blots were visualized by means of chemiluminescence (ECL, Thermo), and the signals were quantified by densitometry. α -Tubulin (analyzed with an antibody from Thermo) served as the loading control.

Levels of interleukin (IL)-1β, IL-6, and tumor necrosis factor-α (TNF-α) in the myocardium or cultured myofibroblasts were assayed with an ELISA (CycLex). Three to five hearts from each group or proteins extracted from myofibroblasts were used for this assay.

### Statistical Analysis

Statistical analysis was performed using SPSS 17.0 software. Values are shown as mean ± SEM. The significance of differences between groups was evaluated with one-way ANOVA followed by the Newman-Keuls multiple comparison test. Values of *P*<0.05 were considered significant.

## Results

### PDE5a Inhibition by Ad-shPDE5a in Cultured Myofibroblasts

**Fig 1:**
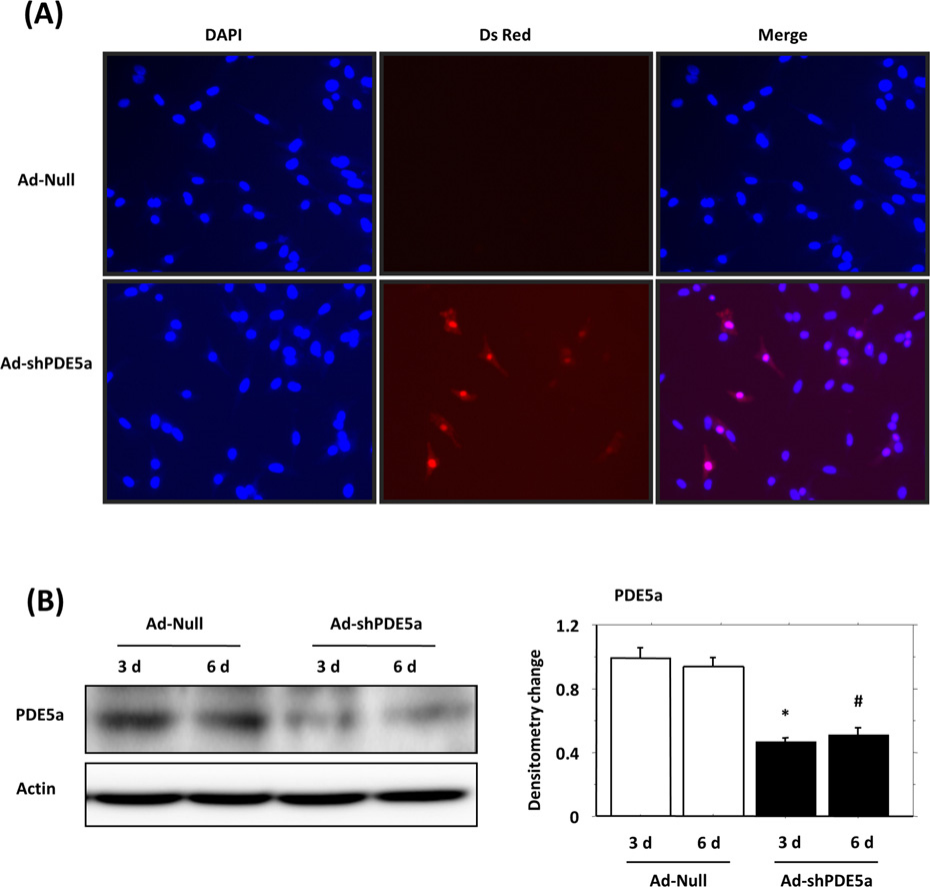
Genetic manipulation of myofibroblasts with Ad-shPDE5a. (A) The cultured myofibroblasts transduced with Ad-shPDE5a (DsRed positive, red fluorescence) at 72 h after transduction. Similar results with relatively low infection rates were obtained more than three times and achieved the desired effect. Ad-shPDE5a treatment successfully inhibits expression of PDE5a *in vivo* and *in vitro*. (B) Western blot showing successful abrogation of PDE5a expression in cultured myofibroblasts observed on days 3 and 6 after transduction with Ad-ShPDE5a. Values plotted in the graph are means ±SEM. *P < 0.05 vs. Ad-Null-treated group on day 3; #P <0.05 vs. Ad-Null-treated group on day 6.

Successful transduction of myofibroblasts with Ad-shPDE5a was performed for abrogation of PDE5a expression (Fig 1A). Western blot showed that 16 h transduction with Ad-shPDE5a significantly inhibited PDE5a expression in the myofibroblasts until days 3 or 6 of observation (Fig 1B).

### Effect of Ad-shPDE5a on Inflammatory Cytokine Expression and Signaling Pathways in Cultured Myofibroblasts

**Fig 2:**
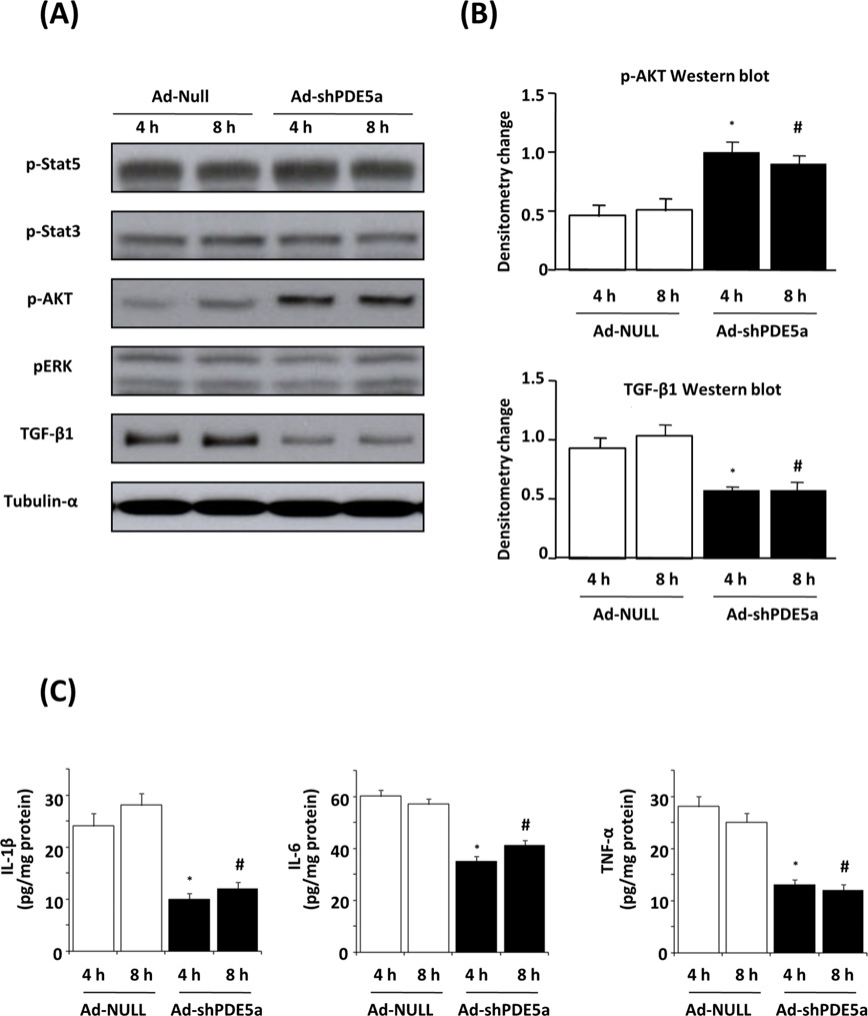
Effect of Ad-shPDE5a on signaling pathways and inflammatory cytokine expression in cultured myofibroblasts. (A) Western blot showing p-Stat3, p-Stat5, p-Akt, p-ERK and TGF-β1expression in Ad-Null or Ad-shPDE5a transduced myofibroblasts cultured at 4 h and 8 h under oxygen and glucose deprivation (OGD). (B) Graphs show TGF-β1 was significantly reduced, and phosphorylated Akt was elevated both at 4 h and 8 h OGD in Ad-Null or Ad-shPDE5a transduced myofibroblasts. *P < 0.05 vs. Ad-Null-treated group on 4 h OGD; #P <0.05 vs. Ad-Null-treated group on 8 h OGD. (C) ELISAs for IL-1β, IL-6 and TNF-α in Ad-Null or Ad-shPDE5a transduced myofibroblasts cultured at 4 h and 8 h under OGD. *P < 0.05 vs. Ad-Null-treated group on 4 h OGD; #P <0.05 vs. Ad-Null-treated group on 8 h OGD.

Western blot analysis showed that myocardial expression of TGF-β1 was significantly reduced, and phosphorylated Akt (but not Stat3/5 or Erk) was elevated 4 h or 8 h after Ad-shPDE5a treatment in cultured myofibroblasts under oxygen and glucose deprivation (Fig 2A and 2B). ELISAs revealed that the levels of IL-1β (10.2±1.1 or 12.1±1.3 pg/mg), IL-6 (35.3±1.9 or 41.2±2.0 pg/mg), and TNF-α (13.1±1.0 or 12.2±1.1 pg/mg) were significantly reduced by Ad-shPDE5a after 4 h or 8 h treatment in cultured myofibroblasts when compared to the IL-1β (24.3±2.5 or 28.1±2.3 pg/mg), IL-6 (60.4±2.3 or 57.3±2.1 pg/mg), and TNF-α (28.2±1.9 or 25.0±1.7 pg/mg) from the Ad-null control (Fig 2C).

### Effects of Ad-shPDE5a Treatment on Cardiac Function and Morphology at Chronic Stage of MI

**Fig 3:**
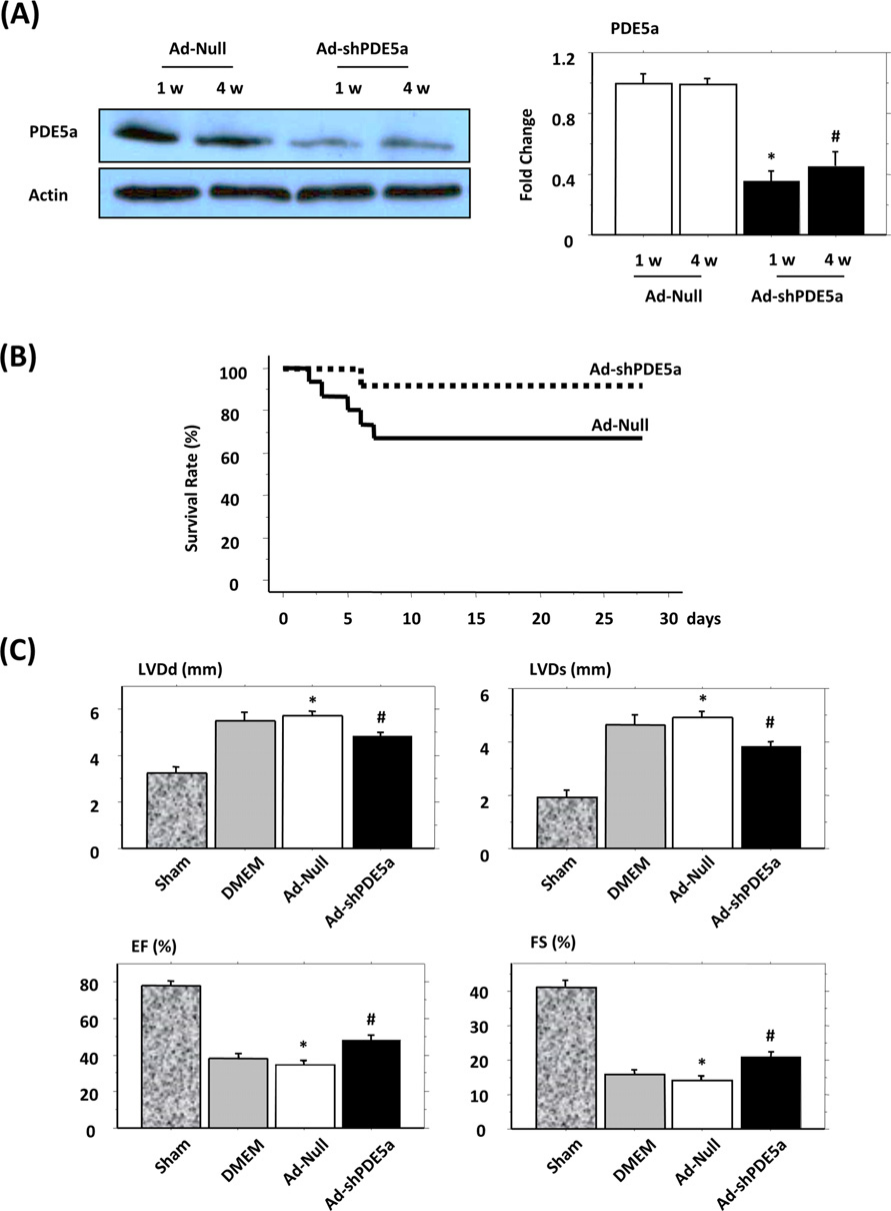
Expression of phosphodiesterase-5a (PDE5a) in vivo, survival curves, and left ventricular (LV) function in various treatment groups of animals. (A) Western blot showing PDE5a expression in the LV of infarcted hearts at 1 and 4 wk after treatment with Ad-Null or Ad-shPDE5a. Graph shows PDE5a levels in Ad-Null treated infarcted hearts were abrogated by Ad-shPDE5a treatment (P <0.01 vs. both Ad-Null groups). (B) Survival curves from Ad-Null and Ad-shPDE5a treatment groups of animals. Compared with 67% in the Ad-Null treated group, the survival rate was 92% in the Ad-shPDE5a-treated group (P = 0.11) (C) echocardiographic data for LV geometry and function 4 wk after treatment with Ad-Null and Ad-shPDE5a. LV diastolic diameter (LVDd), LV end-systolic diameter (LVDs), LV ejection fraction (LVEF), and LV fractional shortening (LVFS) were significantly preserved in Ad-shPDE5a-treated animal hearts. Values are means ± SEM. *P< 0.05 vs. sham operated mice; #P <0.05 vs. Ad-Null-treated mice.

Western blot analysis showed that Ad-shPDE5a treatment significantly decreased PDE5a levels in the infarcted hearts at both 1 wk and 4 wk after treatment compared with Ad-Null-treated animal hearts (Fig 3A). Four wk after coronary artery ligation, 5 (33%) of the Ad-Null, 3 (37%) of DMEM, and 1 (8%) of the AdshPDE5a- treated mice died. The survival rate 4 wk post-myocardial infarction (post-MI) was 67% in Ad-Null-treated and 92% in Ad-shPDE5a-treated animals (p = 0.11, Fig 3B).

Echocardiography carried out 4 wk post-MI revealed that Ad-Null MI mice had marked enlargement of the LV cavity and reduced cardiac function when compared to the sham-operated mice, as indicated by increased LV end-systolic and end-diastolic diameter and reduced LV fractional shortening and ejection fraction (Fig 3C). All of these functional parameters were significantly attenuated in Ad-shPDE5a-treated mice (Fig 3C), suggesting that Ad-shPDE5a treatment mitigated post-MI remodeling and cardiac dysfunction through PDE5a inhibition. There was no significant difference in cardiac function 4 wk post-MI between the Null and DMEM–treated mice, indicating a negligible effect of Null treatment on myocardial function.

**Fig 4:**
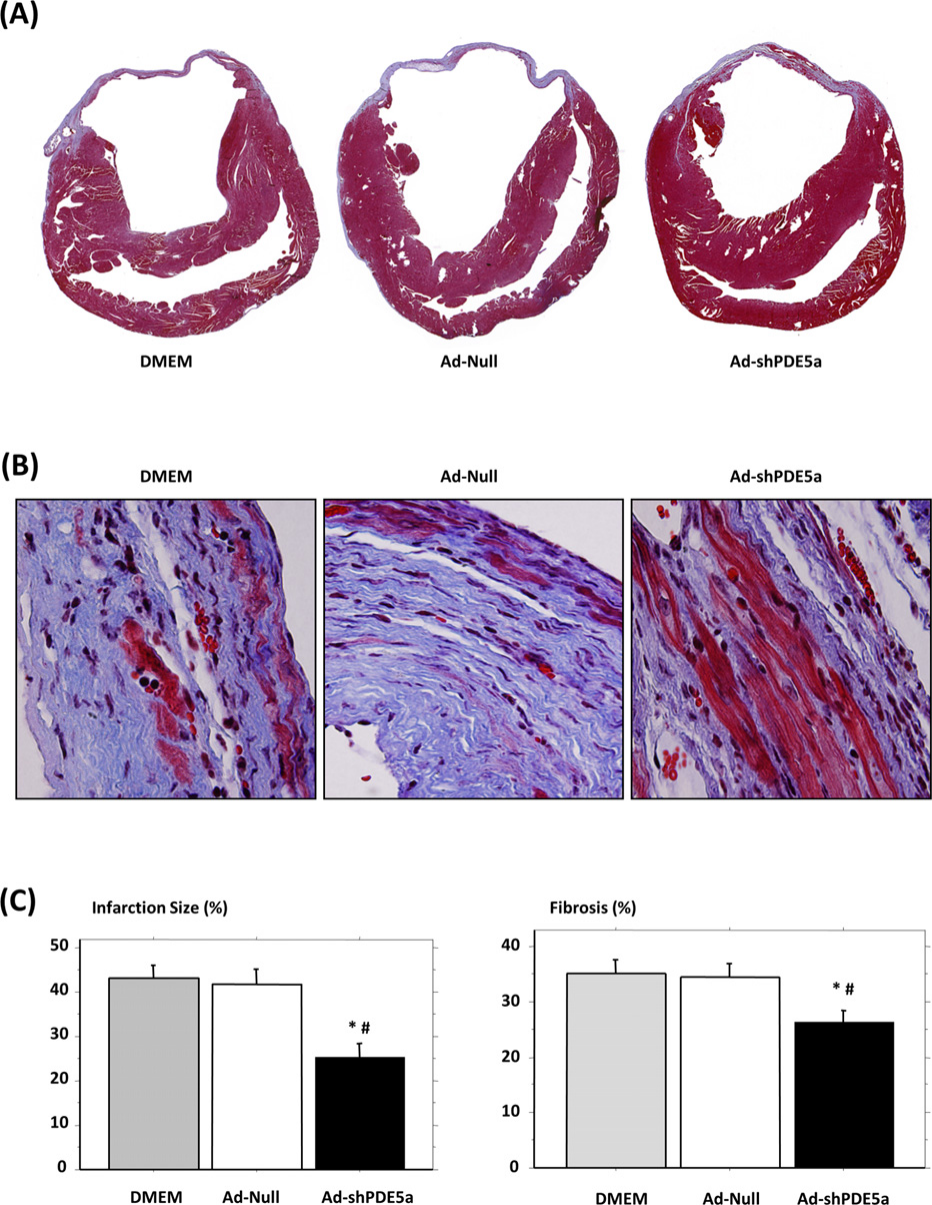
Gross morphology and histology of hearts in different treatment groups of animals at 4 wk post-MI. (A) Transverse ventricular sections of mice hearts treated with DMEM, Ad-Null, and Ad-shPDE5a. Sections were stained with Masson’s trichrome. (B) Residual cardiomyocytes and abundant myofibroblast in the infarcted wall of hearts from different treatment groups stained with Masson’s trichrome. Lesser myofibroblasts were observed in the infarcted wall of Ad-shPDE5a-treated mice. Magnification x1000. (C) Infarct size and fibrosis were significantly reduced in Ad-shPDE5a-treated mice. Values are means ± SEM. *P < 0.05 vs. Ad-Null-treated mice; #P < 0.05 vs. DMEM-treated mice.

Ad-Null or DMEM-treated mice showed marked LV dilatation with a thin infarcted segment 4 wk post-MI, while Ad-shPDE5a-treated mice showed substantially smaller LV cavities and thicker infarcted segments (Fig 4A). Ad-Null and DMEM groups showed disintegrating myocytes within the center of the scar tissue, while abundant viable islands of myocytes were observed in the center of the infarct in the Ad-shPDE5a-treated group (Fig 4B). The size of the infarct was significantly reduced in Ad-shPDE5a-treated animals (25.4±2.9%, P<0.05) compared with Ad-Null (42.0±3.2%) and DMEM groups (43.3±2.6%). Severe fibrosis of the myocardium was observed in the Ad-Null (34.6±2.2) and DMEM groups (35.1±2.5), compared to that of the Ad-shPDE5a treated animals (26.5±1.9, *P*<0.05) (Fig 4C).

### Effect of Ad-shPDE5a on Signaling Pathways and Inflammatory Cytokines Expression in MI Mice

**Fig 5:**
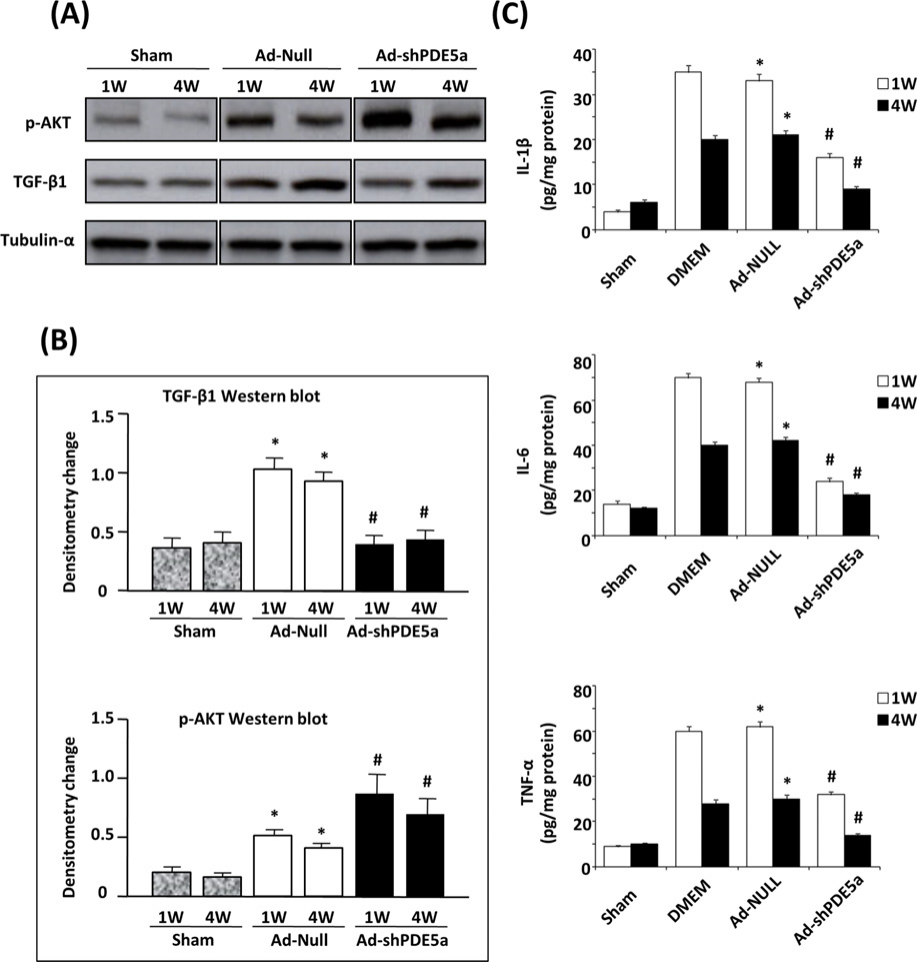
Effect of Ad-shPDE5a on signaling pathways and inflammatory cytokines in MI mice. (A) Western blot shows levels of TGF-β, and phosphorylated Akt in MI hearts at 1 wk and 4 wk after treatment with Ad-Null or Ad-shPDE5a. (B) Graphs show TGF-β1 and p-Akt protein levels were increased in infarcted hearts compared with sham-operated heart. Ad-shPDE5a treatment abrogated the infarction-induced TGF-β1 upregulation as compared to Add-Null, while further increased phosphorylated Akt expression both at 1 wk and 4 wk post-MI. *P <0.05 vs. sham group; #P<0.05 vs. Ad-Null group. (C) ELISAs for IL-1β, IL-6 and TNF-α at 1 wk and 4 wk after DMEM, Ad-Null or Ad-shPDE5a treatment to infarcted hearts *P <0.05 vs. sham group; #P<0.05 vs. Ad-Null group.

Western blot analysis showed that myocardial expression of TGF-β1 and phosphorylated Akt was significantly upregulated in the Ad-Null mice 4 wk post-MI, however, Ad-shPDE5a treatment completely reversed the TGF-β1 upregulation while further increased the expression of phosphorylated Akt (Fig 5A and 5B). ELISAs revealed that the myocardial levels of IL-1β (33.2±1.6 or 21.1±0.9 pg/mg), IL-6 (68.4±2.0 or 42.3±1.7 pg/mg), and TNF-α (62.3±2.1 or 30.1±1.9 pg/mg) were significantly higher in Ad-Null mice at 1 wk or 4 wk post-MI than that of IL-1β (4.1±0.5 or 6.2±0.7 pg/mg), IL-6 (14.0±1.4 or 12.1±0.8 pg/mg), and TNF-α (9.3±0.7 or 10.1±0.6 pg/mg) in sham-operated mice. The elevations were partially or nearly entirely restored to sham levels by Ad-shPDE5a treatment with IL-1β (16.2±1.0 or 9.1±0.6 pg/mg), IL-6 (24.1±1.5 or 18.3±1.1 pg/mg), and TNF-α (32.4±1.4 or 14.2±0.7 pg/mg) (Fig 5C).

## Discussion

Rapid recanalization of the occluded coronary artery is presently the best clinical approach for the treatment of acute MI, but not practical given the small window of therapeutic effectiveness in the few short hours after the onset of MI[19]. Patients who escape death during the acute stage of a large MI are also at high risk of developing heart failure during the later chronic stage [20]. Currently available therapies for post-MI heart failure are underdeveloped and underwhelming, creating great demand for an effective treatment.

PDE5a inhibition prevents the breakdown of cGMP into 5’-GMP that leads to cGMP accumulation. This attribute is currently being exploited as a novel strategy in cardiovascular therapeutics as a method of preventing cardiac remodeling and attenuation of cardiac fibrosis[21]. PKG is a serine/threonine protein kinase and is one of the major intracellular receptors for cGMP. It has been reported that the cGMP/PKG pathway has anti-apoptotic effects on cardiac cells[22]. Accumulation of cGMP in response to PDE5a inhibition counters the ill-effects of adrenergic stimulation and prevents cardiac hypertrophy and pressure-induced remodeling of the heart[23].

RNA interference as an innate biological phenomenon is achieved by loading the RNA interference silence complex with a short single-stranded antisense RNA that is complementary to a target mRNA[24]. For a longer-term inhibition of PDE5a *in vivo*, we constructed a PDE5a-specific shRNA adenoviral vector[11]. Compared with other commercial pharmacological PDE5a inhibitors (Sildenafil, Tadalafil etc.), PDE5a inhibition through Ad-shPDE5a offers the advantage of extended time duration of the protective effects and eliminates the need for repeated treatments.

In the present study, PDE5a enzyme activity was significantly inhibited in the LV after Ad-shPDE5a treatment. This resulted in a significant attenuation of fibrosis, which can contribute to both systolic and diastolic dysfunction[25] subsequent to ischemic episodes. Ad-shPDE5a treatment also mitigated LV remodeling, which was evident from the preserved LV dimensions during both systole and diastole. Finally, Ad-shPDE5a treatment significantly contributed to a reduction in infarction size and preserved myocardial function by maintenance of the viability of the residual cardiac myocytes.

Our previous study provided evidence that post-infarction interference therapy with short hairpin RNA targeting PDE5a relieved the adverse effects on LV geometry and function during the chronic stage[11] of MI. The present study builds on those findings demonstrating that Ad-shPDE5a increases cardiac function and reduces infarction size and fibrosis, revealing that Ad-shPDE5a reduces inflammatory cytokine production in the failing myocardium. Several studies have shown that in both animals and human beings with failing hearts, levels of inflammatory cytokines (IL-1β, IL-6 and TNF- α) are increased in plasma[13] as well as in the myocardium itself[26, 14]. In the present study, levels of IL-1β, IL-6, TNF-α, and TGF-β were elevated in the failing myocardium of mice four weeks post-MI, consistent with those studies. Building upon that, our research revealed that Ad-shPDE5a treatment remarkably decreased the MI-induced inflammatory cytokines: interleukin (IL)-1β, IL-6, tumor necrosis factor-α, and transformed growth factor-β1. Expression of these inflammatory cytokines has been reported to directly relate to the degree of heart failure and relate inversely to survival[14, 27]. Moreover, the results of animal studies and some clinical pilot trials have suggested that suppression of inflammatory cytokines may improve cardiac performance[28, 29]. It is therefore conceivable that reduction of inflammatory cytokines may be one of the mechanisms involved in generating the beneficial effects of Ad-shPDE5a on IM-induced failing hearts.

Phosphatidylinositol-3-kinase (PI3K)/Akt signaling pathway can be activated by a variety of extracellular stimuli and the actions of Akt in the cell are numerous and diverse, but all result in anti-apoptosis or pro-cell proliferation effects[30]. cGMP is a downstream second messenger of nitric oxide (NO). In cardiac myocytes, the physiological effects of cGMP are exerted through the activation of protein kinase G (PKG) signaling. Previous studies have shown that NO inhibits apoptosis of retinal neurons in culture through the canonical cGMP/PKG-dependent pathway, but also involving multiple kinase pathways, such as phosphatidylinositol 3' kinase (PI3k) and Akt [30].Recent studies also showed that the activation of PI3K/Akt is involved in cell survival [31] and axonal outgrowth[32] in neurons. It has been reported that astrocytic Akt phosphorylation might be enhanced through eNOS/sGC/PKG/PI3K pathway, which increased activation of astrocytic Akt may result in the upregulation of pro-survival transcription factor and neuroprotective factor expression[33]. Activated eNOS produces of NO and that NO stimulates soluble guanylate cyclase (sGC), which results in accumulation of cGMP and subsequent activation of the protein kinase G (PKG). The above findings suggest that regulation of PI3K/Akt signaling pathway via activating cGMP/PKG-dependent pathway. This is consistent with our findings that phosphorylated Akt was significantly upregulated in the Ad-Null mice 4-weeks post-MI, and suggests that PDE5a inhibition by Ad-shPDE5a plays a key role in the underlying molecular mechanism for the pro-survival effects, given that AdshPDE5a treatment upregulates Akt signaling pathway via activating cGMP/PKG-dependent pathway. These physiological roles of Akt include involvement in metabolism, protein synthesis, apoptosis pathways, transcription factor regulation, and the cell cycle. Akt exerts its effects in cells by phosphorylating a variety of downstream substrates. Recent studies showed that the activation of PI3K/Akt is involved in cell survival [31] and axonal outgrowth[32] in neurons. These results suggest that NO from eNOS might regulate astrocytic Akt phosphorylation through NO/cGMP/PKG pathway [33]. This is consistent with our findings that phosphorylated Akt was significantly upregulated in the Ad-Null mice 4-weeks post-MI, and suggests that PDE5a inhibition by Ad-shPDE5a plays a key role in the underlying molecular mechanism for the pro-survival effects, given that Ad-shPDE5a treatment upregulates Akt signaling pathway.

## Innovation

PDE5a inhibition with adenoviral short hairpin RNA produced beneficial effects on infarcted mouse hearts that included reduction of infarction size and fibrosis, and an overall improvement of cardiac function and remodeling. The underlying mechanism almost certainly is due in part to activation of the Akt signaling pathway and reduction of inflammatory cytokines, but more research is needed to define that relationship more clearly. These findings indicate that PDE5a inhibition by Ad-shPDE5a could be of significant importance in the design of future therapeutics for post-MI heart failure.
